# Scientific Items

**Published:** 1881-12-20

**Authors:** 


### SCIENTIFIC ITEMS.

**Grafting the Tomato on the Potato.**—Mr. Hiram Stidolph, of Jefferson County, Ala., writing to the Rural World, in October, says: "I have this summer grafted a tomato vine to a potato vine. It is now growing finely. If it had not been such a dry summer I think it would now be full of fruit, and I should probably have potatoes at one end of the vine and tomatoes at the other end. It has been grafted four months and is now (October 15) full of blossoms. It is the most singular piece of grafting I have ever done. I have grafted white currants upon black currants and on red ones, and a gooseberry on a currant, which bore a gooseberry the first year. But grafting the tomato on the potato gave me more trouble than any grafting I ever did. The tomato vine looked sickly for a long time, but I shaded and watered it, and it finally grew and produced blossoms, as I have stated."—*Indianapolis Practitioner.*

**Meteorites.**—What is the origin of these masses of stone and iron? Whence do they come? Various theories have been advanced on the subject, according to which they have been assumed respectively to originate in the atmosphere, to have been ejected from volcanoes, or projected from the moon or some other planetary body. The two former have been dismissed as crude and absurd, and the moon theory, thongh supported by La Place, has fallen into disfavor. Again, they have been supposed to be the *debris* of comets, but the cemmon leaning now seems to be toward Chladni's belief that they are bodies revolving in space, which, coming within reach of the earth's attraction, are precipitated upon it. No theory, however, yet promulgated, accounts for all the known facts. The belief in their terrestial origin occasionally crops up, and is more or less plausibly sustained. Its latest phase as developed by Dr. Ball, Astronomer Royal for Ireland, views them as bodies expelled in the remote past from the earth's volcanoes with velocity sufficient to be carried beyond the earth's immediate attraction and then to move in orbits around the sun. Whenever the earth in its journey round the sun crosses one of the meteroic paths, and one or more of these bodies is present, it is reabsorbed by the earth.

The most recent discovery in connection with this subject is the claim of a German scientist, Dr. Hahn, to have found in stony meteorites indisputable evidence of organic life in the shape of microscopic structures of fossil corals, sponges and sea-weed. Although his claims have some support, it is yet too early to give a definite opinion; but should they eventually be proved true, they will have an important bearing on the question of origin.—*Leffel Mechanical News.*

**A movement** is being pushed among capitalists and builders advocating the use of slate as a substitute for marble and granite in public buildings. The supporters of the movement assert that slate is more durable than marble or granite, and it is impervious to heat. Slate it is said, will absorb the rays of the sun, can be plained, sawed or ground like wood, grows harder by exposure to the weather, and is a building stone that will last for ages. There are four colors of slate—green, red, verigated and purple, and it is proposed to erect a building as a specimen, to be inspected by interested parties.—*Ibid.*

**Snakes.**—M. de Lacerda has found that permanganate of potash is very efficacious as an antidote to the poison of snakes. He experimented on dogs, injecting a one per cent. solution of the substance into the cellula tissue or into the veins, after the poison, and the usual effects of the latter were strikingly obtained. In one series of experiments the poison was allowed time to take some effect before the permanganate was injected, the dogs showing dilation of the pupil, respiratory and cardiac derangements, muscular contractions, etc. Two or three minutes after the antidote was given these troubles disappered, and after fifteen or twenty-five minutes of some measure of prostration, the animal would be able to walk and even run about, and recover its normal aspect. The same dose of poison, not counteracted, caused death, more or less rapidly.—*Ibid.*

**Quicksilver.**—One of the most curious properties of quicksilver is its capability of dissolving or of forming amalgams with other metals. A sheet of gold foil, dropped into quicksilver, disappears almost as quickly as a snowflake when it drops into water. It has the power of separating or of readily dissolving those refractory metals which are not acted upon by our most powerful acids. The gold and silver miners pour it into their machines holding the powdered gold-bearing quartz; and, although no human eye can detect a trace of the precious substance. so fine are the particles, yet the liquid metal will hunt them out, and incorporate it into its mass. By subsequent distillation it yields it into the hands of the miners in a state of virgin purity.—*Ibid.*

**Cold Fire.**—M. Friedel has introduced a new liquid hydrocarbon, which, according to recent experiments, seems to be possessed of extraordinary qualites. It boils at one hundred degrees Fahrenheat, gives a brilliant white light, unaccompanied by heat; and the slightest puff of wind will extinguish it in case of accidental ignition. The corner of a pocket-handkerchief, or even the finger, can be dipped into it, lighted, and used as a temporary torch, without any injury to the novel wick. Owing to the cold produced by the rapid evaporation of the liquid, it would thus seem possible, by means of this new agent, to make one finger serve as a taper whilst sealing a letter with the other.—*Progress of Science.*

**Stain for Mahogany Cherry.**—The most simple and best stain for mahoganizing cherry is ground burnt sienna, mixed in benzine or turpentine. Apply with a brush or sponge, let it stand for a short time, and clean off with a cloth. It will be better to let it remain in this condition until the following day before commencing to finish.—*Druggist and Circular.*

